# Treatment of Aneurism by Compression

**Published:** 1850-11

**Authors:** 


*Treatment of Aneurism by Compression.*—Sir. B. Brodie, at a dinner given to him by the president and council of the Royal College of Surgeons, Dublin (29th August), in returning thanks for the honor done him, said that "It was not then the time to speak of the many improvements in scientific surgery made by some of the Irish surgeons; but he could not refrain from mentioning one of the latest. The mode of curing the formidable disease of aneurism by a bloodless operation, was brought into notice by Irish surgeons, and would, he was convinced, supersede every other plan."

